# The Importance of User Interface and User Experience in the Adoption of Whole Slide Images for Clinical Use

**DOI:** 10.1155/2014/836176

**Published:** 2014-12-14

**Authors:** Christopher Garcia

**Affiliations:** Department of Pathology, Massachusetts General Hospital, Boston, MA 02114-2696, USA

## Introduction

The Emphasis of User Experience (UX) in software design has greatly contributed to the recent evolution of user interface. As consumer software and web applications become more intuitive, it is important for new software solutions entering the workspace to be more intuitive as well.

## Methods

The Google Android Design principles are an example of UX design principles that focus on the entire experience of the user. The main principles of “Enchant Me,” “Simplify My Life,” and “Make Me Amazing” are applied and examined in the current user interfaces present in WSI software: case navigation, slide navigation, slide analysis, and annotation.

## Results

Each user interface present in WSI software is developed and normalized to different extents across different vendor solutions. Case navigation is the least developed and the least standardized, while also being the interface that demonstrates the least application of UX principles. WSI navigation and analysis/annotation are both more mature in that they are fairly uniform across vendor software. However, analysis shows that the application of UX principles in both interfaces is immature.

## Conclusion

Principles of UX could be used for dramatic effect on WSI user interface in bettering the experience of pathologists as they continue to adopt whole slide imaging software for clinical needs. As the experience becomes more intuitive and less cumbersome, adoption may be made more willingly.

